# Mental Health Consequences of the COVID-19 Pandemic Among Ontario’s Youth: A Cross-Sectional Study

**DOI:** 10.7759/cureus.22526

**Published:** 2022-02-23

**Authors:** Muhammad A Hamid, Aljeena Rahat Qureshi, Suruchi Kapoor, Wardha Shabbir, Atchaya Arulchelvan, Manasvi Vanama, Farwa Abdi, Luxhman Gunaseelan

**Affiliations:** 1 Pediatrics, University of Toronto, Toronto, CAN; 2 Health Sciences, McMaster University, Hamilton, CAN; 3 Internal Medicine, Kozikode Medical College, Kozikode, IND; 4 School of Medicine, Windsor University Medical School, Cayon, KNA; 5 Medicine, University of Toronto, Toronto, CAN; 6 Health Sciences, University of Waterloo, Toronto, CAN; 7 Health Sciences, York University, Toronto, CAN; 8 Internal Medicine, Saba University School of Medicine, Devens, USA

**Keywords:** pediatrics, obsessive compulsive disorder, anxiety, depression, covid-19

## Abstract

The coronavirus disease 2019 (COVID-19) pandemic has had a significant impact on the mental health and wellbeing of Ontario's youth. Our study investigated the psychological impacts of COVID-19 on the pediatric population of Ontario, using a survey derived from the Revised Children's Anxiety and Depression Scale (RCADS) system to identify children who may benefit from seeking professional help. Our cross-sectional study examined the potential risk factors that contributed to worsening mental health and wellbeing in children, including changes in sleep patterns, appetite, and physical activity levels, as well as the diagnosis of a family member with COVID-19. Our study found that 24%, 9.4%, and 15.5% of participants exhibited symptoms of depression, anxiety, and obsessive-compulsive disorder (OCD), respectively, according to the RCADS system. Furthermore, there were significant associations between the presence of symptoms and the diagnosis of a family member with COVID-19 or a frontline worker in the family. This suggests a need to create interventions to support the families of frontline workers and those directly affected by a COVID-19 diagnosis.

## Introduction

The coronavirus disease 2019 (COVID-19) pandemic has had a significant impact on the mental health of people across the whole world, including Ontario's youth [1]. The transition to online schooling, as well as the lockdowns and social distancing requirements, have led to significant lifestyle changes that may have had a significant impact on the mental health and wellbeing of Ontario’s pediatric population. There have been multiple case studies reported in the US and all parts of the world demonstrating the worsening of mental health conditions, including depression, anxiety, post-traumatic stress disorder (PTSD), and obsessive-compulsive disorder (OCD) [2-3]. For children, in particular, social isolation, excessive screen time, and a lack of socialization were associated with profound effects, resulting in mental health conditions such as major depressive disorder, generalized anxiety disorder, PTSD, and OCD [4].

This is highlighted in a recent study by McArthur et al., where 846 mothers and children participated in a COVID-19 mental health questionnaire in Calgary, Canada [4]. The study identified an increase in the reported number of anxiety and depression symptoms such as connectedness to caregivers, child sleep, and screen time duration.

Another longitudinal study by Bignardi et al. that assessed childhood depression symptoms during the pandemic found that there was a significant increase in depression symptoms as a result of the lockdown in the UK [5]. Mental health assessments, including self-reports, parent reports, and teacher reports, were taken before and during the pandemic in 168 children between the ages of seven and 12. The study reported greater child-reported RCADS depression (r=−0.08, 95% CI −0.16 to 0.01) and anxiety (r=−0.09, 95% CI −0.16 to 0.01) symptoms during the pandemic than before the pandemic [5].

Our study aimed to investigate the psychological impacts of COVID-19 on the pediatric population of Ontario, using a survey completed by parents. Although a survey is not an appropriate diagnostic tool for mental health conditions, our survey uses the Revised Children's Anxiety and Depression Scale (RCADS) system to identify children who may benefit from seeking professional help. Our cross-sectional study examined the potential risk factors that contribute to worsening mental health and wellbeing in children, including changes in sleep patterns, appetite, and physical activity levels, as well as the diagnosis of a family member with COVID-19.

## Materials and methods

Our study is a cross-sectional study in which we asked the parents of children in grades 3 to 12 of all races and genders to fill out an anonymous online survey investigating any possible development of symptoms of anxiety, depression, and obsessive-compulsive disorder during the COVID-19 pandemic. Furthermore, our survey asked questions pertaining to noticeable behavioral changes in the child concerning aspects such as physical activity, sleep, and appetite. Children were excluded from the study if they had psychological or physical comorbidities prior to the beginning of the COVID-19 pandemic. Parents were asked to provide informed consent on behalf of the child prior to filling out the survey. Anonymity was preserved through a lack of personal identifying information. Our survey was a standardized survey that was derived from the Revised Children’s Anxiety and Depression Scale (RCADS) that is available for use at no cost, given in a Likert scale, and designed using Cognito Forms (Columbia, SC) (Appendix 1). A poster with an easily accessible link and QR code that took parents to the survey form was used to recruit participants (Appendix 2). The poster was shared on various social media sites including Facebook and Instagram. Research Ethical Board approval was obtained through the Scarborough Health Network (IRB approval #: PED-21-013). The data collection period was July 8 to October 8, 2021, and we were able to recruit 246 children in total.

To contextualize this time period, by early July in 2021, the province had seen continued improvement in key public health and health care indicators such as hospitalizations, ICU occupancy, and the weekly cases incidence rates [6]. On July 16, 2021, the province moved into step three of the roadmap to reopen in which the operation of many indoor services with larger numbers of people and restrictions in place was resumed, such as outdoor social gatherings and organized public events with up to 100 people with limited exceptions [6]. Face coverings in indoor public settings and physical distancing requirements remained [6]. This remained in place until October 25, 2021, after which Ontario lifted capacity limits, including physical distancing requirements in most settings [7].

The demographic characteristics of the participants are presented in Table 1. After the completion of data collection, the data were transferred from Cognito Forms to Microsoft Excel (Microsoft Corporation, Redmond, WA) in order to generate T-score values using the RCADS module for symptoms of depression, anxiety, and OCD. Correlating risk factors and the T-Scores associated with the psychiatric condition gave us further insight into the psychological impact of the COVID-19 pandemic on Ontario’s pediatric population. Chi-square tests for depression, anxiety, and OCD against gender, family COVID-19 diagnosis, and family frontline worker were performed to determine the significance of any associations between these variables and the development of symptoms.

**Table 1:** Characteristics of study participants OCD: obsessive-compulsive disorder

	n (n=246)	%
Gender		
Male	130	52.9
Female	116	47.2
Grade		
3	35	14.2
4	31	12.6
5	22	8.9
6	15	6.1
7	22	8.9
8	29	11.8
9	18	7.3
10	31	12.6
11	16	6.5
12	27	11
Personal/family diagnosis of COVID-19		
Yes	40	16.3
No	206	83.7
Frontline worker in the family		
Yes	100	40.7
No	146	59.4
Depression positive	59	24
Anxiety positive	23	9.4
OCD positive	38	15.5
	Mean	SD
Total depression score	8.78	7.49
Total anxiety score	5.11	5.19
Total OCD score	4.47	4.97

## Results

A total of 246 children participated in this study. The majority of participants were male (52.9%), in Grade 3 (14.23%), had no personal or family COVID-19 diagnosis (83.74%), and had no frontline workers in the family (59.35%).

Depression symptoms

Twenty-four percent (24.0%; n=59) of participants were found to be positive for symptoms of depression using the RCADS clinical cut-off of T-scores over 65. As shown in Table 2, there was a significant difference in the cases of depression between those that had a personal or family diagnosis of COVID-19 (X^2^ (n = 59) = 49.358, p = 0.014) or a frontline worker in the family (X^2^ (n = 59) = 45.004, p = 0.039). The difference in depressive symptoms between genders was nonsignificant (X^2^ (n = 59) = 36.549, p = 0.191).

**Table 2:** Chi-square test results for symptoms of depression *: p < 0.05, a: Adjusted for age

Variable	Depression (n=59)		
	n (%)	X^2^ (dof=30)^a^	p-value
Gender			
Male	27 (45.8)	36.549	0.191
Female	32 (54.2)		
Diagnosis of COVID-19 in the family			
Yes	12 (20.3)	49.358	0.014*
No	47 (79.7)		
Frontline worker in the family			
Yes	34 (57.6)	45.004	0.039*
No	25 (42.4)		

Anxiety symptoms

Among study participants, 9.4% (n=23) developed symptoms of anxiety using the RCADS clinical cut-off of T-scores over 65. The presence of a personal or family diagnosis of COVID-19 (X^2^ (n = 23) = 52.733, p = 0.000) or a frontline worker in the family (X^2^ (n = 23) = 35.600, p = 0.017; see Table 3) was found to be associated with anxiety symptoms. The difference in symptoms of anxiety between genders was nonsignificant (X^2^ (n = 23) = 26.403, p = 0.153) (Table 3). 

**Table 3:** Chi-square test results for symptoms of anxiety *: p < 0.05, a: Adjusted for age

Variable	Anxiety (n=23)		
	n (%)	X^2^ (dof=20)^a^	p-value
Gender			
Male	7 (30.4)	26.403	0.153
Female	16 (69.6)		
Diagnosis of COVID-19 in the family			
Yes	5 (21.7)	52.733	0.000*
No	18 (78.3)		
Frontline worker in the family			
Yes	11 (47.8)	35.600	0.017*
No	12 (52.2)		

OCD symptoms

Fifteem point five percent (15.5%; n=38) of participants had symptoms of anxiety. Significant associations were found between anxiety symptoms and the presence of a personal or family diagnosis of COVID-19 (X^2^ (n = 38) = 55.596, p = 0.000) and a frontline worker in the family (X^2^ (n = 38) = 48.453, p = 0.000; see Table 4). There was no significant association between gender and symptoms of OCD (X^2^ (n = 38) = 20.919, p = 0.191) (Table 4).

**Table 4:** Chi-square test results for symptoms of OCD *: p < 0.05, a: Adjusted for age OCD: obsessive-compulsive disorder

Variable	OCD (n=38)		
	n (%)	X^2^ (dof=18)^a^	p-value
Gender			
Male	28 (73.7)	20.919	0.191
Female	10 (6.32)		
Diagnosis of COVID-19 in the family			
Yes	13 (34.2)	55.596	0.000*
No	25 (65.8)		
Frontline worker in the family			
Yes	20 (52.6)	48.453	0.000*
No	18 (47.4)		

## Discussion

This cross-sectional study aimed to assess the impact of the COVID-19 pandemic on Ontario’s pediatric population by examining potential risk factors. We discovered that there were significant differences in the development of children’s symptoms of depression, anxiety, and OCD when associated with the child having family members working on the frontline or if there was a positive COVID-19 diagnosis in the family. Gender was not a significant risk factor for the development of anxiety, depression, or OCD symptoms in children during COVID-19.

Our findings are consistent with other studies that also suggest the rise of youth mental health difficulties during the COVID-19 pandemic [8]. This may be attributed to restrictions on peer interactions, reduced contact with teachers and other supports, social isolation, and lack of access to school support for mental health services [9-10]. These results have physiological implications as well-the worsening of mental health conditions in children has been shown to also contribute to worsening physical health symptoms such as pain [11].

Cost et al. examined COVID-19 exposure as a potential risk factor for pediatric mental health concerns [12]. However, a seminal finding from our study is the difference between frontline workers’ families and non-frontline workers’ families in the development of anxiety, depression, and OCD in Canadian pediatric samples. Frontline healthcare workers have been found to be among the most vulnerable groups at risk of mental health concerns, particularly in the COVID-19 pandemic setting. Temsah et al. found that while there were no COVID-19 cases reported yet in Saudi Arabia at the time of data collection, healthcare workers reported significantly higher anxiety levels from COVID-19 as opposed to the Middle East respiratory syndrome coronavirus (MERS-CoV) or seasonal influenza [13]. Frontline workers, including physicians, have been found to be at a higher risk for suicide during the pandemic as well [14]. Similarly, many Canadian frontline workers report high rates of depression, anxiety, and insomnia, and over 70% report psychological distress [15-16]. However, the downstream effects on children of frontline worker families due to their high risk of infection, increased work stress, and fear of spreading the infection to their families, have yet to be investigated [17]. To our knowledge, this is the first Canadian study suggesting such a relationship.

The association found between the development of pediatric mental health disorders and the chronic and acute stressors associated with frontline work is alarming, suggesting a heightened need to create effective interventions to help frontline workers cope with and adequately support their children and families. Perhaps this is indicative of a weakness in the Canadian response to the pandemic. Efforts must be made by workplaces when implementing occupational health measures to look beyond people’s function as frontline responders and to adopt a more holistic approach that takes into account their societal roles as parents, spouses, and offspring. A study among Moroccan healthcare workers explores the effects of how 40% of healthcare workers marry each other, causing a “childcare crisis” that causes disruption to traditional solidarity systems, potentially exposing children of healthcare workers to additional emotional challenges caused by the absence of both parents [18].

This prompts one to consider the state of childcare for frontline workers in Canada. As of March 17, 2020, licensed child care centers were closed, aside from select locations to support health care and other frontline workers. Then, from June 12, 2020, child care centers were allowed to reopen throughout the province [19]. It was only until April 15, 2021, that fees for childcare were waived for eligible frontline workers [20]. Further research on the effectiveness of the current child care system and interventions to mitigate the burden on frontline worker families on an institutional level is warranted.

On the other hand, the findings of our study may hint at something greater than an infrastructure issue such as parents being ill-equipped to face the novel challenges posed by parenting due to the pandemic. In a few studies, it was shown that a rise in parental stress levels is associated with harsher parenting while perceived control over stressful environments while supportive family environments act as a buffer to decrease stress [21-22]. COVID-19 has been accompanied by an unprecedented range of stressors threatening the well-being of families. Brown et al. have found a positive association between parental perceived stress, greater COVID-19-related stressors, high anxiety, and depressive symptoms [23]. Families with frontline workers and those exposed to a COVID-19 diagnosis face additional stressors to parenting due to the nature of their work. Even the long-term impacts of telework for non-essential workers on the relationships between parents and children is unknown. Could the pandemic have improved familial relationships as individuals begin to spend more time together or worsen relationships such as in the case of toxic or abusive homes? This suggests that interventions that aim to bolster parenting education, such as culturally responsive whole-family programs and services with a focus on the parent-child relationship, warrant further study [24].

This study has several limitations. The study design was cross-sectional, preventing us from making causal inferences on the relationship between frontline worker families, families with a positive COVID-19 diagnosis, and symptoms of pediatric mental health disorders. Longitudinal research is required to evaluate these potential risk factors and capture their effect in light of changing COVID-19 restrictions. The RCADS scale used in this study while a validated screening tool for symptoms of depression, anxiety, and OCD cannot ascertain cases with certainty. A clinician needs to be present to review each individual case in order to make a diagnosis. Another limitation of the study was that the survey collected limited data regarding participant characteristics, preventing a more holistic examination of sociodemographic risk and protective factors for the development of pediatric mental health concerns. Additionally, while advertisements were shared in networks serving Ontarians and participants were required to self-identify as Ontario residents, it cannot be said with certainty that all respondents were from Ontario exclusively. While the results were consistent with the general literature, our findings cannot be broadly generalized. Finally, our survey was only administered in English, which excludes the experiences of non-English-speaking parents in Ontario. However, as of 2016, only 2.5% of Ontarians were found to be non-English speaking so perhaps these results may still be generalizable to the Ontario population [25].

Despite these limitations, our findings illustrate the important associations of COVID-19 diagnosis exposure, frontline workers within families, and pediatric mental health disorders. Our findings have widespread implications for prevention and intervention programming such as developing the infrastructure to provide frontline workers with more flexibility and access to childcare supports. This may include increasing flexibility for workers with families and reducing barriers to accessing care for these vulnerable populations such as subsidizing transportation costs and expanding no-cost emergency childcare programs. In addition, the findings imply that parents may benefit from public health messaging and improved family-centered healthcare that better equips them with the difficulties of raising children in the context of a global pandemic. Finally, our study suggests that there is a gap in the literature regarding the effects of occupational stress on frontline workers' and their families' well-being, warranting further research.

## Conclusions

In conclusion, this study examined the psychological impact of COVID-19 diagnosis exposure and family members who are frontline workers on a pediatric sample in Ontario. Institutions with frontline workers and parents will benefit from knowing about the potential downstream impacts of occupational stress on children and adolescents. In addition, public health and education agencies may benefit from a greater understanding of stressors affecting children, allowing integration into intervention programs. The current research provides preliminary insight into the myriad of factors affecting pediatric individuals.

## Figures and Tables

**Figure 1:**
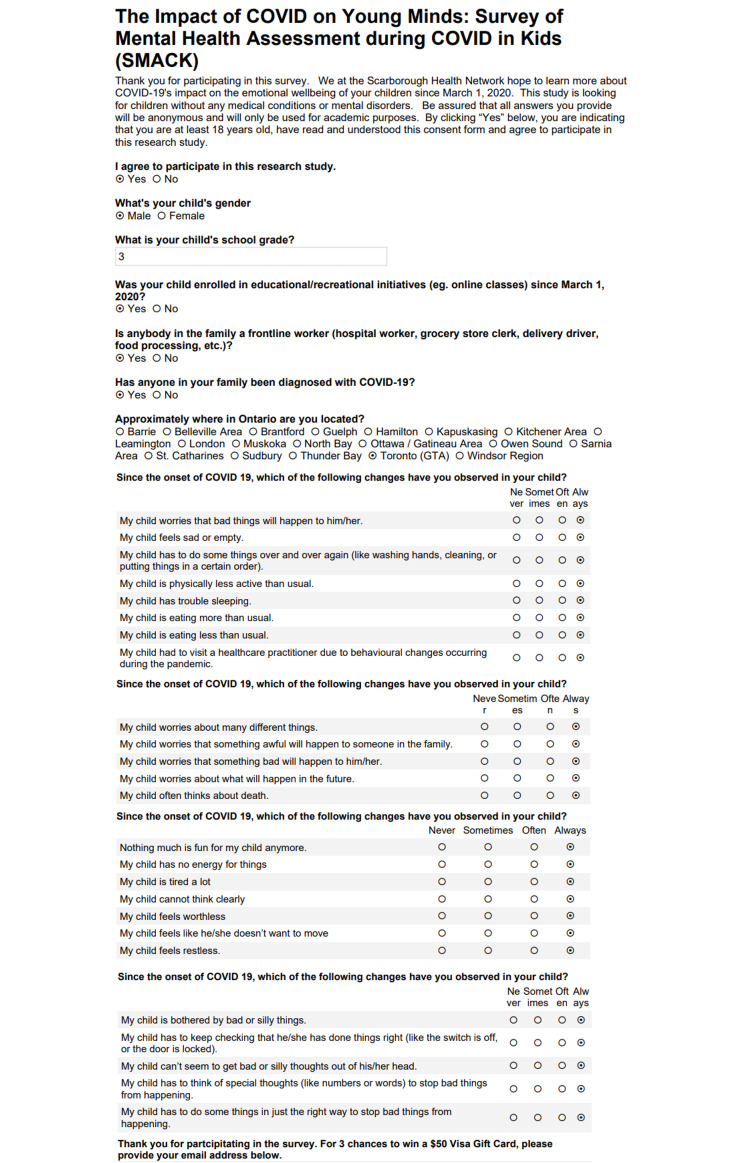
Survey used in the study, distributed through online platforms via. Cognito Forms

**Figure 2:**
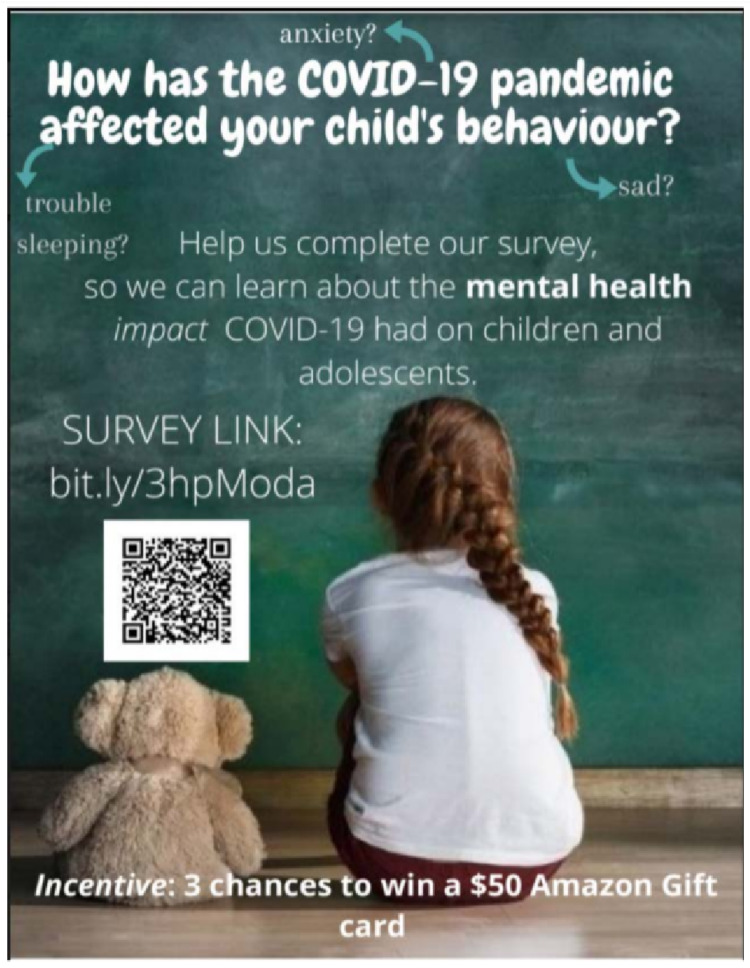
Poster used to recruit survey participants
